# Gut-spilling in chordates: Evisceration in the tropical ascidian *Polycarpa mytiligera*

**DOI:** 10.1038/srep09614

**Published:** 2015-04-16

**Authors:** Noa Shenkar, Tal Gordon

**Affiliations:** 1 Zoology Department, George S. Wise Faculty of Life Sciences, Tel-Aviv University, Tel-Aviv, Israel

## Abstract

The ejection of internal organs, i.e., evisceration, is a well-known phenomenon in sea-cucumbers. We report the ability of a member of the Chordate phyla, the tropical ascidian *Polycarpa mytiligera*, to eviscerate and regenerate its gut within 12 days, and to rebuild its branchial sac within 19 days. Evisceration occurred within 4–43 seconds of gentle mechanical pressure exerted on the tunic in 47% of the tested *P. mytiligera*. Individuals were able to discard up to 3/4 of their digestive tract via the incurrent siphon by rupture of the branchial sac in this area. Although chemical analysis revealed no significant levels of toxic compounds, the eviscerated guts were unpalatable to the triggerfish and pufferfish on which they were tested, suggesting evisceration as a defense mechanism. Given the close affinity of ascidians to vertebrates, the regeneration pathway of the viscera and branchial sac of ascidians suggests its potential beneficial application in soft tissue regeneration research.

The wide range of regenerative powers within the animal kingdom has drawn the attention of scientists since the early 18^th^ century^1^. From hydras to planarians and geckos, the ability of certain species to redevelop various parts of the body and regain some or all of their original form and function presents fundamental opportunities for research in the fields of cell signaling, development, and adaptation. Holothurians (sea-cucumbers) are well-known for their ability to regularly discard completely or mostly their internal organs in response to an external stimulus^2^, or on a seasonal basis^3^. The enormous progress that has been made in the field of holothurian evisceration research in the past two decades has revealed important molecular mechanisms^4^, cellular pathways^5^, cancer-related gene expression^6^,^7^, and organogenesis processes^8^,^9^, thus establishing a solid ground for studies on post-traumatic regeneration^10^. Although the regenerative powers of ascidians, a unique group of filter-feeder marine organisms belonging to the Deuterostomes, have been studied extensively, this has focused mainly on the regeneration of colonial species^11^, and partial body-part regeneration of the siphonal region following artificial amputation^12^. However, in the late 19^th^ century scientists documented several different species of solitary ascidians lacking their viscera and/or the branchial sac (Table S1). Sluiter (1885)^13^, even named one of the species that lacked both the branchial sac and the gut - *Styeloides abranchiata*. Wiley (1897)^14^ commented that in another closely-related species, which he named *Styeloides eviscerans*, the animals survive the evisceration procedure, suggesting that regeneration of the eviscerated body parts (referring mainly to the branchial sac) occurs from the endostyle^15^, a deep ciliated groove along the ventral-mid line of the branchial sac which secrets mucus. Despite the fascinating opportunities to study evisceration in ascidians, this phenomenon was only anecdotally mentioned by taxonomists in the following decades in several other species (see Supplementary Table S1), leaving basic questions unanswered, such as: How are the viscera ejected? Does the animal survive following evisceration? Does it rebuild its viscera; and if so - how? Our documentation of evisceration response of the solitary ascidian *Polycarpa mytiligera* (Savigny, 1816), a conspicuous member on Indo-Pacific coral reefs^16^,^17^, provides a unique opportunity to deepen our knowledge and revive the study of evisceration in ascidians, establishing a solid platform from which to study regeneration of the digestive tube from molecular, cellular, and developmental aspects.

## Results

**Figure 1:**
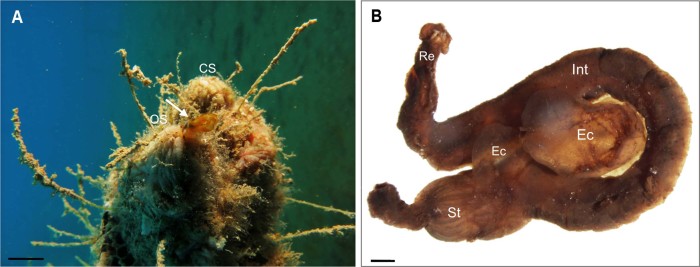
Evisceration in the solitary ascidian *Polycarpa mytiligera*, Gulf of Aqaba, Red Sea. (a) Evisceration in the field, arrow pointing to the gut expelled through the oral siphon (OS). CS- cloacal siphon. Scale bar 1 cm. (b) Eviscerated gut, including the stomach (St), intestine (Int), endocarp (Ec, a projection of the body wall into the atrial cavity), and part of the rectum (Re). Scale bar 1 mm. Photo: G. Koplovitz, T. Gordon.

**Figure 2:**
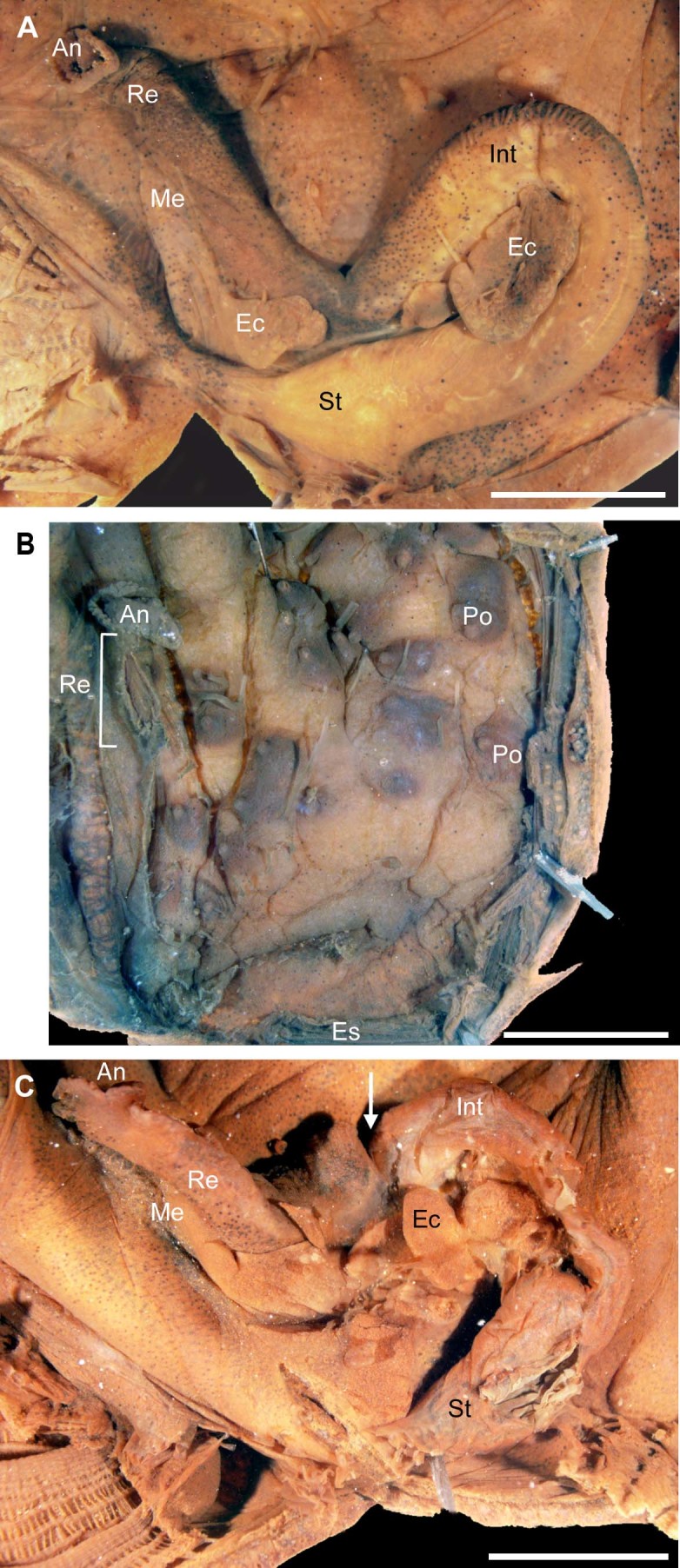
*Polycarpa mytiligera* digestive tract comparisons. (a) Complete digestive tract of an undisturbed animal. Voucher specimen AS25769 (b) digestive tract region following evisceration. Voucher specimen AS25770 (c) regenerated digestive tract 12 days post-evisceration, arrow pointing to the area of amputation of the old gut. Scale bar 5 mm. Stomach (St), intestine (Int), endocarps (Ec), rectum (Re), anus (An), membrane connecting the rectum to the body wall (Me), polycarps (Po, gonads), endostyle (Es). Voucher specimen AS25771. Photo: N. Shenkar.

**Figure 3:**
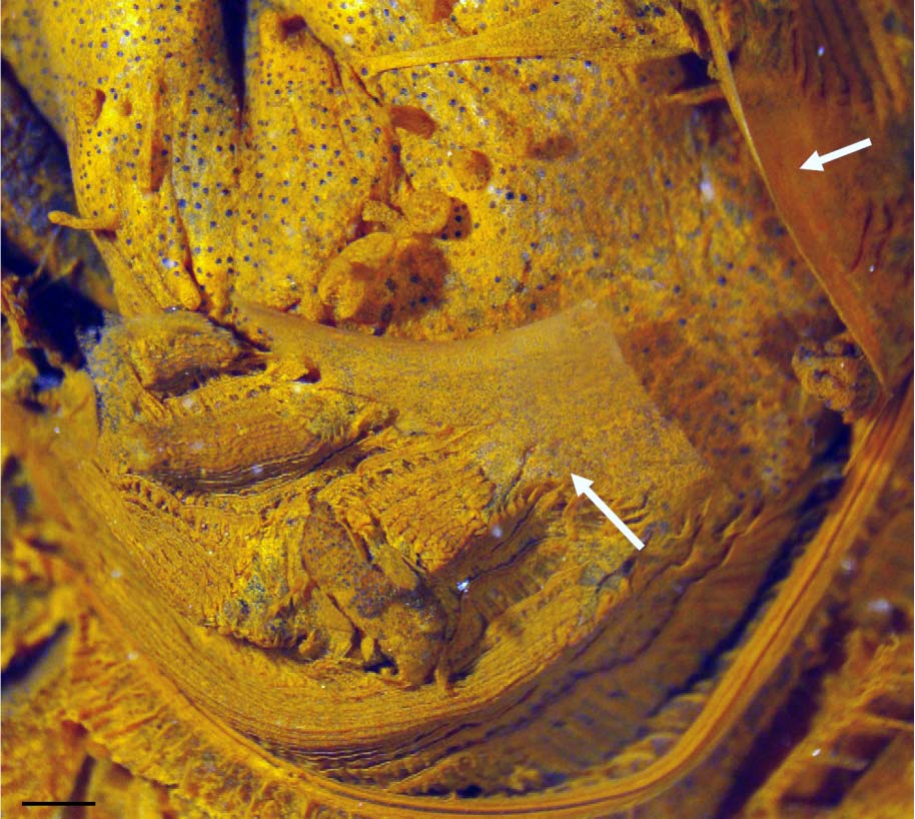
*Polycarpa mytiligera* regeneration of the branchial sac. Arrows pointing to the area of new tissue formation five days post-evisceration. Scale bar 1 mm, Voucher specimen AS25772. Photo: N. Shenkar.

*Polycarpa mytiligera* is a relatively large species (about 6 cm in length) with a tough brownish tunic covered by epibionts. Following gentle squeezing of the tunic it is able to eviscerate its gut through the incurrent opening (oral siphon, Fig. 1, video S1). We found that this phenomenon occurred in 47% of our attempts to stimulate evisceration (n = 66 different individuals), leaving the eviscerated animals in the field in a highly contracted state, with both siphons closed. Marked eviscerated individuals that were followed daily in the field, and individuals that were induced to eviscerate in water tables, demonstrated the same reaction following evisceration: 1–2 days of highly contracted bodies with siphons completely closed, followed by 3–4 days with siphons partially open and a minor reaction to an external stimulus (n = 4). One week post-evisceration the animals demonstrated a healthy external appearance of wide-open siphons and a normal rapid contraction following an external stimulus. The average length of the eviscerated gut was 2.5 ± 1.04 cm (n = 18), which is about 1/2 of the entire intestinal loop (4.7 ± 0.8 cm, n = 20). By sampling eviscerated individuals from the field at weekly intervals, we were able to follow the progress of the branchial sac and gut regeneration, examine the physiological state of the animal, and follow the cascade of events leading to expulsion of the gut. The primary gut loop in individuals that were not induced to eviscerate is attached to the test with only 2–3 trabeculae, while the long rectum, which forms an acute angle with the primary loop, is attached firmly to the test by a continuous membrane (Fig. 2a). Thus, the eviscerated component of the digestive tube is usually composed of the gut loop, including the stomach, and a large endocarp (a projection of the body wall into the atrial cavity) attached to the gut, leaving the animal with a torn branchial sac on the left side, and an oesophagus and rectum (Fig. 2b). Morphological examination of eviscerated individuals revealed that while there is definite damage to the branchial sac, which is ripped in the region above the primary gut loop, there is no damage to the adjacent gonads (termed polycarps, Fig. 2b). Twelve days after evisceration, specimens were found with a completely new gut with fecal pellets and a mucus thread in the branchial sac, implying active filter-feeding. The new guts were small and narrow, enclosing a number of small regenerating endocarps (Fig. 2c). They were loosely attached to the body wall with 1–3 trabeculae, and contained digested material. The branchial sac regeneration occurred from the margins of the ruptured area, and had reached completion after only 19 days post-evisceration (Fig. 3). The evisceration response occurs swiftly, with an average duration of 16 ± 13.5 sec (n = 7, calculated from video-clips) from initial contact. Chemical analysis of the eviscerated gut revealed only regular levels of heavy metals and other toxic elements (see Supplementary Table S2). However, a variety of unfed aquaria fish such as triggerfish and pufferfish (n = 11) that were offered the eviscerated guts consistently ejected them after initially swallowing (see Supplementary Table S3).

## Discussion

Prior to the observations provided here, evisceration in ascidians has been sporadically noted by ascidian taxonomists as an abnormal phenomenon. The current study is the first to demonstrate this behavior as a natural response to mechanical stress in a solitary tropical ascidian. *Polycarpa mytiligera* is highly abundant on hard surfaces in coral reefs, on which strong-jawed fish such as triggerfish and pufferfish often predate various benthic invertebrates. Thus, it is possible that *P. mytiligera*, which is camouflaged by its epibiont growth, will be accidently bitten, a stimulus resulting in evisceration of the gut in order to either distract the predator fish, or signal the ascidian's unpalatability. Following evisceration the animal remains in a highly contracted state, which together with its massive epibiont cover provides complete camouflage against an additional attack (video S1). Although ascidians are well known for their ability to accumulate heavy metals^18^, the chemical analysis of the eviscerated gut did not reveal any significant amounts of toxic elements. Yet, our feeding assays revealed an absolute rejection by the tested fish of the eviscerated guts (Table S3), indicating an alternative strategy of *P. mytiligera* by which to induce a repellant response, possibly by secondary metabolites^19^. Predation pressure is a key factor in shaping tropical ecosystem assemblages^20^. Thus, evolving such a defensive trait provides a strong advantage, possibly contributing to the high abundance of *P. mytiligera* in this habitat.

The evisceration response through the incurrent siphon leaves the animal with a torn branchial sac (figure 3). The branchial sac is a chamber perforated by dorso-ventral rows of gill slits called stigmata, which are formed during the juvenile stages^21^. Stigmata development has been studied in several species since they are considered as key structures for the understanding of the evolution of the deutrostome body plan^22^,^23^. Our documentation of the gradual regeneration and recovery of the branchial sac provides researchers with a unique insight into the development of the stigmata in adult individuals, and to the formation of the branchial folds in Stolidobranch ascidians.

The fascinating ability of solitary ascidians such as *P. mytiligera* to completely regenerate their digestive system and repair the branchial sac within a period of less than three weeks, provides a unique insight into the regenerative powers within the Chordate phyla. In view of the growing recognition of the potential of marine organisms to contribute to the treatment of post-traumatic injuries in humans^24^,^25^,^26^, findings from the study of the evisceration phenomenon in ascidians, which have a close affinity to vertebrates^27^, are expected to contribute to our understanding of the treatment of visceral injuries in humans.

## Methods

### Field and laboratory observations

In order to determine whether the animals survive following evisceration, we artificially induced evisceration in four individuals of *P. mytiligera* occuring at 10 m depth in front of the Inter-University-Institute (IUI) in Eilat, Red-Sea (29°30'06.2″N 34°55'01.7″E) in October 2013. Two of these individuals were marked in the field with a plastic wire and monitored on a daily basis for the first week of study, and on a weekly basis following one month post-evisceration. The other two individuals were transferred to the IUI and maintained in a water table with running seawater after being attached with glue to a ceramic plate. These individuals were monitored on a daily basis for one month.

In order to ascertain how common is the evisceration phenomenon, we attempted to induce evisceration artificially by gently squeezing 66 individuals overgrowing the bottom of a floating dock in Eilat, Red Sea (29°32'51.93″N 34°57'13.47″E) in November 2013. The mechanical pressure was maintained until the animal had either expelled its gut, or for no longer than one minute. Only one attempt was conducted on each individual. To quantify latency to evisceration and to follow the expulsion path of the gut, we photographed and analyzed video-clips of each attempt using a Canon Powershot G15 camera in an Ikelite underwater housing.

### Regeneration progress experiment

In November 2013, we marked with tagged plastic wires 12 individuals that had been induced to eviscerate in the field. The discarded gut length of these 12 specimens was estimated by analyzing gut photos using ImageJ image analysis software^28^. Five gut samples of the largest size group were immediately transferred to −20°C for future ICP-MS chemical analysis. Of the marked individuals in the field, we sampled three individuals weekly and preserved them in 4% formaldehyde solution^17^. Dissected specimens were studied and photographed using a Nikon SMZ18 stereomicroscope. All specimens are deposited at The Steinhardt Museum of Natural History and National Research Center at Tel-Aviv University (voucher numbers AS25769-AS25810).

### Heavy metal analysis

The eviscerated gut samples were digested in a Milestone Ethos 1600 microwave, using 70% HNO_3_, 30% HCl and 30% H_2_O_2_ in Teflon vessels. The analysis was done using Agilent 7700 ICP-MS by Milouda and Migal Laboratories, Israel.

### Feeding assays

In order to observe the reaction of coral-reef fish to the eviscerated gut, we ran several feeding trials at the underwater observatory facilities in Eilat. The trials were conducted in February 2014, using five large aquaria that the fish inhabit as part of the regular exhibition. Thus, the fish were well acclimated to this environment and are used to being hand-fed by the staff. We used a number of common strong-jawed fish such as triggerfish and pufferfish (24 individuals total, Table S3) which were kept unfed for a period of 24 hours. The guts were obtained earlier that day by inducing evisceration in 20 *P. mytiligera* individuals, and kept on ice until introduced into each aquarium. At each feeding trial we introduced one gut in a similar way to that in which the fish are fed regularly; hence, the fish were able to notice the gut in the water, and we followed their reactions using video. We conducted three attempts in each aquarium, using a new gut each time, and introducing only complete digestive tubes of similar size. Each trial lasted for three minutes, and fish reaction was categorized as: no reaction, in which the fish did not approach the gut piece; swallow and spit; swallow and ingest. To determine whether the fish response was due to lack of hunger, following the feeding trials the fish were offered their regular brine shrimp mix, which they all accepted (100% of individuals investigated).

## Supplementary Material

Supplementary Information Supplementary tables

Supplementary Information Supplementary video

## References

[b1] Smith J. & Olds J. L. Models and mechanisms of regenerative biology across phylogeny: introduction to a virtual symposium in The Biological Bulletin. Biol. Bull. 221, 3–5 (2011). 21876106 10.1086/BBLv221n1p3

[b2] Emson R. & Wilkie I. Fission and autotomy in echinoderms (Aberdeen University Press, 1980).

[b3] Swan E. F. Seasonal evisceration in the sea cucumber, *Parastichopus californicus* (Stimpson). Science 133, 1078–1079 (1961). 17742774 10.1126/science.133.3458.1078

[b4] Sun L., Yang H., Chen M., Ma D. & Lin C. RNA-Seq reveals dynamic changes of gene expression in key stages of intestine regeneration in the sea cucumber *Apostichopus japonicas*. PloS one 8, e69441 (2013). 23936330 10.1371/journal.pone.0069441 PMC3735544

[b5] García‐Arrarás J. E. *et al.* Cellular mechanisms of intestine regeneration in the sea cucumber, *Holothuria glaberrima* Selenka (Holothuroidea: Echinodermata). J. Exp. Zool. 281, 288–304 (1998). 9658592 10.1002/(sici)1097-010x(19980701)281:4<288::aid-jez5>3.0.co;2-k

[b6] Mashanov V. S., Zueva O. R. & Garcia-Arraras J. E. Expression of *Wnt9*, *TCTP*, and *Bmp1/TII* in sea cucumber visceral regeneration. Gene Expr. Patterns 12, 24–35 (2012). 22079950 10.1016/j.gep.2011.10.003 PMC3272084

[b7] Mashanov V. S., Zueva O. R., Rojas-Catagena C. & Garcia-Arraras J. E. Visceral regeneration in a sea cucumber involves extensive expression of survivin and mortalin homologs in the mesothelium. BMC Dev. Biol. 10, 117 (2010). 21114850 10.1186/1471-213X-10-117 PMC3013081

[b8] Mashanov V. & García-Arrarás J. Gut regeneration in holothurians: a snapshot of recent developments. Biol. Bull. 221, 93–109 (2011). 21876113 10.1086/BBLv221n1p93

[b9] Pasten C., Rosa R., Ortiz S., González S. & García-Arrarás J. E. Characterization of proteolytic activities during intestinal regeneration of the sea cucumber, *Holothuria glaberrima*. Int. J. Dev. Biol. 56, 681 (2012). 23319344 10.1387/ijdb.113473cp PMC4068352

[b10] Mashanov V. S., Zueva O. & Garcia-Arraras J. E. Postembryonic organogenesis of the digestive tube: why does it occur in worms and sea cucumbers but fail in humans? Curr. Top. Dev. Biol. 108, 185–216 (2013). 10.1016/B978-0-12-391498-9.00006-1 PMC5432039 24512710

[b11] Kürn U., Rendulic S., Tiozzo S. & Lauzon R. J. Asexual propagation and regeneration in colonial ascidians. Biol. Bull. 221, 43–61 (2011). 21876110 10.1086/BBLv221n1p43

[b12] Jeffery W. R. Closing the wounds: one hundred and twenty five years of regenerative biology in the ascidian *Ciona intestinalis*. Genesis 53, 48–65 (2015). 24974948 10.1002/dvg.22799 PMC4277945

[b13] Sluiter C. P. Ueber einige einfachen Ascidien von der Insel Billiton. Natuurk. Tljdschr. Ned. Indie. 45, 160–232 (1885).

[b14] Willey A. Letters from New Guinea on Nautilus and some other organisms. QJ Microsc. Sci. N. Ser 39, 145–180 (1897).

[b15] Selys-Longchamps M. D. Autotomie et regeneration des visceres chez Polycarpa tenera Lacaze et Delage. Compte Rendu de l'Academie des Sciences Paris 160, 566–569 (1915).

[b16] Monniot F. & Monniot C. Ascidians from the tropical western Pacific. Zoosystema 23, 201–376 (2001).

[b17] Shenkar N. Ascidian (Chordata, Ascidiacea) diversity in the Red Sea. Mar. Biodiv. 42, 459–469 (2012).

[b18] Carlisle D. & Bernal J. Vanadium and other Metals in Ascidians [and Discussion]. P. Roy. Soc. Lon. B. Biol. 171, 31–42 (1968). 10.1098/rspb.1968.0054 4385858

[b19] Pisut D. P. & Pawlik J. R. Anti-predatory chemical defenses of ascidians: secondary metabolites or inorganic acids? J. Exp. Mar. Biol. Ecol. 270, 203–214 (2002).

[b20] Freestone A. L., Osman R. W., Ruiz G. M. & Torchin M. E. Stronger predation in the tropics shapes species richness patterns in marine communities. Ecology 92, 983–993 (2011). 21661559 10.1890/09-2379.1

[b21] Garstang W. On the Development of the Stigmata in Ascidians. Proc. R. Soc. A. 51, 505–513 (1892).

[b22] Manni L., Lane N. J., Zaniolo G. & Burighel P. Cell reorganisation during epithelial fusion and perforation: the case of ascidian branchial fissures. Dev. Dyn. 224, 303–313 (2002). 12112460 10.1002/dvdy.10112

[b23] Shimazaki A., Sakai A. & Ogasawara M. Gene expression profiles in *Ciona intestinalis* stigmatal cells: insight into formation of the ascidian branchial fissures. Dev. Dyn. 235, 562–569 (2006). 16342199 10.1002/dvdy.20657

[b24] Addad S., Exposito J.-Y., Faye C., Ricard-Blum S. & Lethias C. Isolation, characterization and biological evaluation of jellyfish collagen for use in biomedical applications. Mar. drugs 9, 967–983 (2011). 21747742 10.3390/md9060967 PMC3131555

[b25] Bermueller C. *et al.* Marine collagen scaffolds for nasal cartilage repair: prevention of nasal septal perforations in a new orthotopic rat model using tissue engineering techniques. Tissue Eng. Pt. A 19, 2201–2214 (2013). 10.1089/ten.tea.2012.0650 PMC3762606 23621795

[b26] Mariottini G. L. & Pane L. Cytotoxic and cytolytic cnidarian venoms. A review on health implications and possible therapeutic applications. Toxins 6, 108–151 (2013). 24379089 10.3390/toxins6010108 PMC3920253

[b27] Delsuc F., Brinkmann H., Chourrout D. & Philippe H. Tunicates and not cephalochordates are the closest living relatives of vertebrates. Nature 439, 965–968 (2006). 16495997 10.1038/nature04336

[b28] Schneider C. A., Rasband W. S. & Eliceiri K. W. NIH Image to ImageJ: 25 years of image analysis. Nat. Methods 9, 671–675 (2012). 22930834 10.1038/nmeth.2089 PMC5554542

