# Supplementary material for: Gut-spilling in chordates: Evisceration in the tropical ascidian Polycarpa mytiligera
Source: Sci Rep. 2015 Apr 16;5:9614. doi: 10.1038/srep09614 (PMC5381747; doi:10.1038/srep09614)
Supplement: Supplementary Information — Supplementary tables [file srep09614-s1.doc]

**Title:** Gut-spilling in chordates: Evisceration in the tropical ascidian *Polycarpa mytiligera*

**Authors:** Noa Shenkar^1^* and Tal Gordon^1^

**Supplementary material**

Video S1

Tables S1-S3

References (#29-37)

**Table S1. Evisceration records of ascidians.**

| **Species** | **Locality** |
| --- | --- |
| *Polycarpa captiosa* | Philippines ^16^, South Africa^29^ |
| *Polycarpa cryptocarpa* | Melanesia ^30^, New Caledonia^31^ |
| *Polycarpa gradata* | Mozambique^32^ |
| *Polycarpa longiformis* | Australia^33^ |
| *Polycarpa madagascariensis* | South Africa^29^ |
| *Polycarpa nigricans* | New Caledonia^31^ |
| *Polycarpa olitoria* | Australia^33,34^, South Africa^29^ |
| *Polycarpa papillata* | Australia^33^, Gulf of Manaar^35^, Indonesia^13^, New Caledonia^31^, Philippines^36^, South Africa^29,37^ |
| *Polycarpa rubida* | Mozambique^29^ |
| *Polycarpa tenera* | Roscoff^15^ |
| *Styeloides evisscerans** | New Guinea^14^ |

*Unaccepted name

**Supplement references**

29 Monniot, F., Monniot, C., Griffiths, C. L. & Schleyer, M. South African ascidians. *Ann. S. Afr. Mus.* **108**, 1-141 (2001).

30 Tokioka, T. Ascidians collected during the Melanesia Expedition of the Osaka Museum of Natural History. I, Ascidians presented by Dr. R. L. A. Catala of the Aquarium of Noumea. *Publs Seto Mar. Biol. Lab*. **9(1)**, 104–138 (1961).

31 Monniot, C. Ascidies de Nouvelle-Calédonie. II. Les genres *Polycarpa* et *Polyandrocarpa*. *Bulletin du Muséum National d’Histoire Naturelle, Paris*. **9A**, 275–310 (1987).

32 Monniot, C. Stolidobranch ascidians from the tropical western Indian Ocean. *Zool. J. Linn. Soc.* **135.1**, 65-120 (2002).

33 Kott, P. The Australian Ascidiacea. Part. I. Phlebobranchia and Stolidobranchia. *Mem. Qd Mus*. **23**, 1–440 (1985).

34 Sluiter, C. P. Tunicaten. In: Semon, R, ed. Zoologische Forschungsreisen in Australien und den Malagischen Archipel. *Jenaische Denkschriften.* **8**, 163- 186 (1895)**.**

35 Herdman, W .A. Report on the Tunicata. *Ceylon Pearl Oyster Fisheries*  *Suppl.* **39**, 295–348 (1906).

36 Tokioka, T. Ascidians from Mindoro Island, The Philippines. *Seto Mar. Biol.*

*Lab.* **18(2)**, 75-107 (1970).

37 Sluiter, C .P.  Beiträge zur Kenntnis der Fauna von Südafrica Ergebnisse einer Reise von Prof. Max Weber in Jahre 1894. II. Tunicaten von Süd Africa. *Zool.*  *Jb. Abtheilung für Systematik, Geographie und Biologie der Thier*. **11**, 1–64 (1898).

**Table S2. Heavy metal content in *Polycarpa cryptocarpa*** eviscerated gut, n=5

| **Voucher number** | **AS25771** | **AS25773** | **AS25774** | **AS25775** | **AS25776** |
| --- | --- | --- | --- | --- | --- |
| **Sample weight (g)** | 0.0952 | 0.069 | 0.042 | 0.896 | 0.115 |
| **Metal (mg/kg)** |  |  |  |  |  |
| **Li** | <0.01 | 0.56 | <0.01 | 1 | 0.47 |
| **Be** | <0.004 | <0.01 | <0.01 | <0.004 | <0.003 |
| **B** | 26.61 | 7.86 | 29.83 | 26.05 | 44.53 |
| **Mg** | 2579 | 2392 | 2777 | 3153 | 2977 |
| **Al** | 77.35 | 665.67 | 294.11 | 521.94 | 294.25 |
| **Ti** | 4.82 | 53.79 | 19.47 | 32.95 | 12.17 |
| **V** | 0.44 | 4.27 | 0.83 | 3.91 | 1.8 |
| **Cr** | <0.002 | 2.28 | 0.24 | 2.06 | 0.65 |
| **Mn** | 2.59 | 24.61 | 5.73 | 20.69 | 8.66 |
| **Fe** | 90.49 | 844.58 | 202.46 | 793.92 | 274.76 |
| **Ni** | 0.8 | 1.95 | 0.52 | 1.82 | 0.86 |
| **Cu** | 2.82 | 3.74 | 3.13 | 5.55 | 2.56 |
| **Zn** | 31.64 | 58 | 29.29 | 34.73 | 26.42 |
| **As** | 1.37 | 1.73 | 1.05 | 1.94 | 1.19 |
| **Se** | 0.4 | 0.31 | 0.18 | 0.46 | 0.27 |
| **Sr** | 18.2 | 87.1 | 27.22 | 75.07 | 65.1 |
| **Mo** | <0.04 | <0.051 | <0.07 | <0.04 | <0.09 |
| **Ag** | <0.003 | <0.004 | 0.08 | 0.01 | 0.38 |
| **Cd** | <0.001 | <0.002 | <0.002 | <0.001 | <0.001 |
| **Sn** | <0.002 | <0.002 | <0.003 | <0.002 | <0.001 |
| **Sb** | <0.004 | <0.01 | <0.01 | <0.004 | <0.003 |
| **Ba** | 2.51 | 5.38 | 1.72 | 7.19 | 6.93 |
| **Pb** | <0.001 | <0.001 | <0.001 | <0.001 | >0.005 |

**Table S3. Feeding assay experiment.** Species investigated, number of individuals and response. N = number of different individuals.

| **Response** | **N** | **Species** | **Aquarium** |
| --- | --- | --- | --- |
| swallow and spit | 1 | *Rhinecanthus aculeatus* | 1 |
| swallow and spit | 1 | *Balistapus undulatus* |
| swallow and spit | 2 | *Amphiprion bicinctus* |
| no reaction | 1 | *Rhinecanthus aculeatus* | 2 |
| no reaction | 1 | *Sufflamen albicaudatum* |
| swallow and spit | 2 | *Zebrasoma xanthurum* |
| no reaction | 1 | *Arothron hispidus* | 3 |
| no reaction | 1 | *Rhinecanthus aculeatus* |
| swallow and spit | 1 | *Sufflamen albicaudatum* |
| swallow and spit | 1 | *Zebrasoma xanthurum* |
| swallow and spit | 1 | *Bodianus axillaris* |
| no reaction | 1 | *Arothron hispidus* | 4 |
| swallow and spit | 1 | *Rhinecanthus aculeatus* |
| swallow and spit | 1 | *Arothron diadematus* |
| no reaction | 1 | *Pseudobalistes flavimarginatus* |
| swallow and spit | 1 | *Scolopsis ghanam* |
| no reaction | 5 | *Ostracion cubicus* | 5 |
| no reaction | 1 | *Canthigaster margaritata* |
